# Case Report: ALCAPA syndrome: successful repair with an anatomical and physiological alternative surgical technique

**DOI:** 10.12688/f1000research.8823.2

**Published:** 2016-08-03

**Authors:** Luis Gustavo Vilá Mollinedo, Andrés Jaime Uribe, José Luis Aceves Chimal, Roberto Pablo Martínez-Rubio, Karen Patricia Hernández-Romero

**Affiliations:** 1 Cardiothoracic Surgery Department, National Medical Centre 20 de Noviembre, ISSSTE, Mexico City, Mexico

**Keywords:** Coronary vessel anomalies, ALCAPA syndrome, Bland-White-Garland syndrome, Adults with ALCAPA, Coronary extension technique

## Abstract

Anomalous left coronary artery from the pulmonary artery, or ALCAPA syndrome, is a rare congenital cardiac disease that can cause myocardial infarction, heart failure and even death in paediatric patients. Only few untreated patients survive until adult age. Here we present the case of a 33-year-old female patient with paroxysmal tachycardia, syncope and mild exertional dyspnoea. She was diagnosed with ALCAPA syndrome and underwent surgical correction with an alternative technique of left main coronary artery extension to the aorta.

## Introduction

ALCAPA syndrome, also known as Bland-White-Garland Syndrome, is a rare congenital heart disease, affecting approximately 1 of 300,000 newborns in the USA. If left untreated 90% of patients die during the first year of life, due to myocardial ischemia and heart failure. Approximately 18–25% of patients with this congenital heart disease reach adulthood, presenting arrhythmias, heart failure and myocardial ischemia ^1^. Treatment of the anomalous origin of the left coronary artery from the pulmonary artery includes several surgical techniques, however they are all associated with increased morbidity (21%) ^2– 5^.

In the adult, surgical correction is burdensome, due to the heart dimensions and compensatory disorders in coronary circulation to the left ventricle. We present the case of a 33-year-old female with ALCAPA syndrome and mitral valve severe regurgitation, who underwent successful correction with a physiological and anatomical technique.

## Clinical case

**Figure 1.**
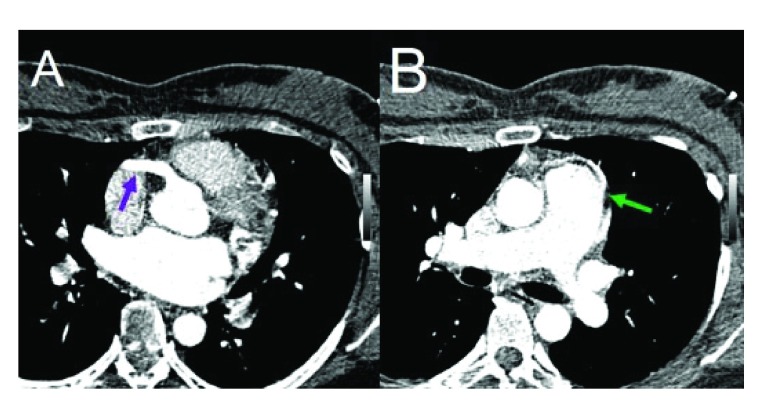
**A** – Arrow – RCA dilated arising from the aorta. **B** – Arrow – LMA arising from lateral aspect of the MPA.

A 33-year-old female with medical history of recurrent respiratory infections since childhood, paroxismal tachicardia in adolescence and some syncope episodes in adulthood accompanied by retrosternal pain during exercise. Physical examination revealed a mitral murmur III/IV. The paraclinical diagnostic methods exhibited an anomalous emergency of the left main trunk from the pulmonary artery, the right coronary dilated, the left ventricle dilated and regurgitant flow in mitral valve ( Figure 1).

## Surgical technique and postoperative follow-up

**Figure 2.**
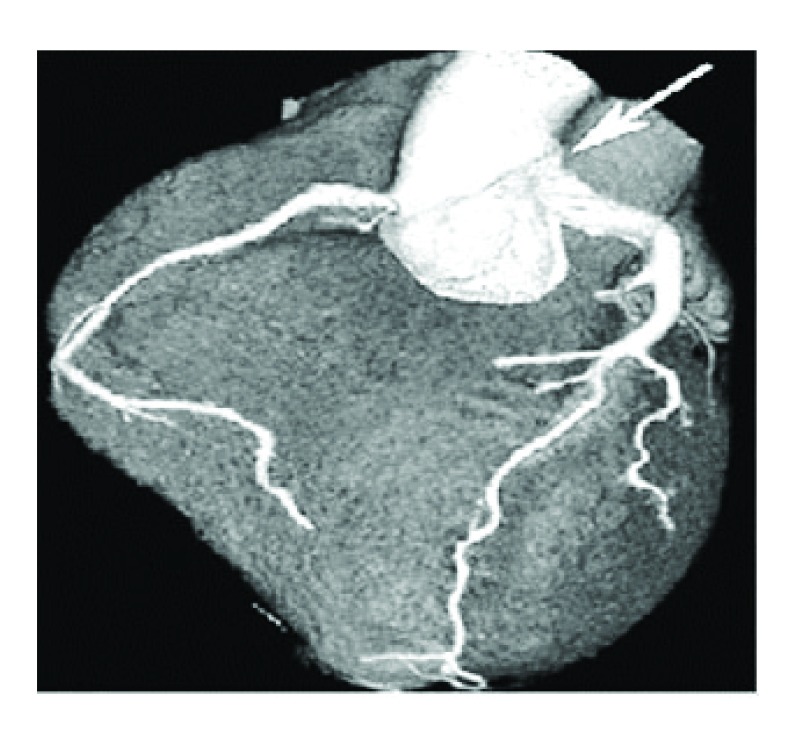
AngioCAT – Arrow – Adequate graft patency (combined pulmonary tissue and bovine pericardium patch).

Sternotomy and surgical procedure were performed with circulatory support to hypothermia (28°C). The mitral valve was replaced by Mechanical Sorin Carbomedics *®* valve No 27 to correct valvular dysplasia. The left main coronary artery button was dissected and then connected to a duct constructed with pulmonary wall and bovine pericardium to be anastomosed to the aorta artery. The pulmonary artery was reconstructed with Woven Dacron graft, leaving the previously constructed duct in the back of the Dacron graft. The surgical findings were: right coronary (RCA) dilated and collateral circulation from RCA to left ventricular circulation, LMA arising from the MPA, dysplasia of posterior mitral valve. At 6 months follow-up, the patient remained in functional class I of New York Heart Association and AngioCAT showed patency of the new ductus ( Figure 2).

## Discussion

ALCAPA congenital anomaly is a rare disease that must be surgically treated in the first year of life. However, between 10–15% of patients reach adulthood and clinically manifest rhythm disorders usually attributed to alterations of the cardiac electrical system, which obscures the underlying pathophysiology of myocardial ischemia ^1– 5^.

The blood flow restauration in left main coronary artery from the aorta is the primary objective in the surgical correction of ALCAPA, and there are several surgical options in the paediatric population. Derivation of the left subclavian artery and implementation of an aorto-coronary bypass with saphenous vein or the left internal thoracic artery to the left anterior descending coronary have shown low short-term effectiveness (60%) and high morbidity with stenosis and thrombosis of bypass graft ^10– 14^. The Takeuchi procedure is the most used in the paediatric population, however it has a high incidence (> 21%) of supravalvular stenosis of pulmonary artery ^15^.

**Figure 3.**
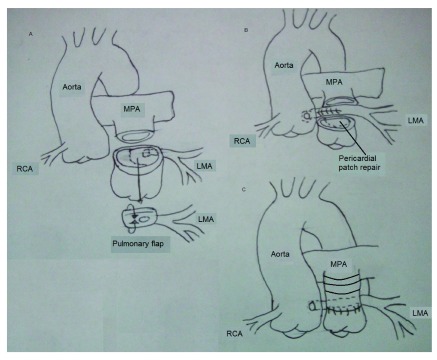
**A** – LMA taken from the MPA and reconstructed as a tubular structure with bovine pericardium. **B** – LMA anastomosis to the Ao as in a normal position, MPA reconstructed with a pericardial patch. **C** – MPA reconstructed with a Dacron graft.

In the first year of life, great arteries are not fully developed and tissues are more “flexible”, which allows a coronary reimplantation. However, child’s growth promotes stenosis in short and medium-term ^7– 9^. The major anatomical distances and the less “flexible” tissues in adult patients make the surgical restauration of the left main coronary artery blood flow more difficult. Our surgical team resolved this situation with a duct constructed with pulmonary artery wall (80%) and bovine pericardial patch (20%), leaving this duct in anatomical position behind the Woven dacron graft used for restitution of blood flow in main pulmonary artery ( Figure 3). We believe that anatomical position of the new duct permits a physiologic blood flow like in a normal heart. In our case, ischemic symptoms resolved and the patient maintained good functional class at 6 months follow-up and full patency of the graft in AngioCAT.

**Figure 4.**
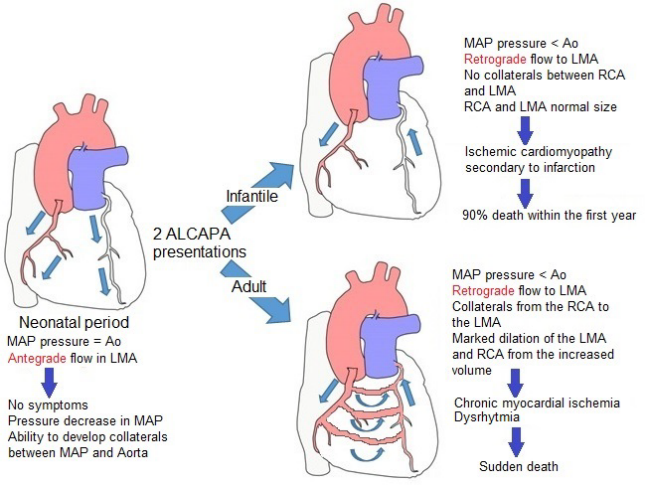
ALCAPA syndrome pathophysiology.

The ALCAPA pathophysiology consists of a relative coronary steal, which promotes low oxigenation in the left myocardial tissue as a consequence of blood flow from pulmonary artery which leads to myocardial ischemia and acute myocardial infarction. The low oxygenation circumstance promotes collateral vessels development and right coronary dilatation, as can be seen in Figure 4. On the other hand, the chronic myocardial ischemia produces papillary muscle and ventricular lateral wall dysfunction, which causes mitral insufficiency. All of this would explain the symptoms presented by the patient.

Mitral insufficiency treatment is still under discussion; some authors prefer valvular reconstruction, considering that the failure is due to papillary muscle dysfunction; nevertheless, an important proportion of insufficiency recurrence still exists. In a sense other authors prefer to replace the mitral valve with a valvular prosthesis. In the case that we presented, the surgical team observed a valve dysplasia which prevented valvular reconstruction, so it was decided to replace the mitral valve with a mechanical prosthesis.

To summarise, the ALCAPA or Bland-White-Garland syndrome treatment is a real surgical challenge in the adult population. However, we believe that the alternative procedure presented in this article consisting of pulmonary artery wall and bovine pericardial construction of a new duct, which connects the left main coronary artery re-establishing a normal anatomical situation and permitting a physiological blood flow to left ventricle, are a viable and probably successful surgical alternative in adult patients without risk of pulmonary stenosis.

## Consent

Written informed consent for publication of their clinical details was obtained from the patient.
