# Supplementary material for: Case Report: ALCAPA syndrome: successful repair with an anatomical and physiological alternative surgical technique
Source: F1000Res. 2016 Aug 3;5:1680. Originally published 2016 Jul 13. [Version 2] doi: 10.12688/f1000research.8823.2 (PMC4975369; doi:10.12688/f1000research.8823.2)
Supplement: Peer review history

# Peer review: Referee response for version 2

**DOI:** 10.5256/f1000research.10067.r15415

**Published:** 2016-08-05

**Authors:** Aiden Abidov

**Affiliations:** 1 Department of Medicine and Radiology, University of Arizona College of Medicine, Tucson, AZ, USA

Excellent report - all previous concerns were successfully addressed in the revised version.

 No further comments.

I have read this submission. I believe that I have an appropriate level of expertise to confirm that it is of an acceptable scientific standard.

# Peer review: Referee response for version 2

**DOI:** 10.5256/f1000research.10067.r15414

**Published:** 2016-08-04

**Authors:** Stephan Achenbach

**Affiliations:** 1 Department of Internal Medicine II (Cardiology), University of Erlangen, Erlangen, Germany

I have read this submission. I believe that I have an appropriate level of expertise to confirm that it is of an acceptable scientific standard.

# Peer review: Referee response for version 1

**DOI:** 10.5256/f1000research.9498.r14947

**Published:** 2016-07-26

**Authors:** Stephan Achenbach

**Affiliations:** 1 Department of Internal Medicine II (Cardiology), University of Erlangen, Erlangen, Germany

There is nothing wrong with the description of a surgical technique for ALCAPA repair in the form of a case report. This, however is no proof of superiority over other approaches.

 The main concern is presentation:

 “ALCAPA syndrome, also known as Bland-White-Garland Syndrome, is a rare congenital heart disease, affecting approximately 300,000 newborns in the USA.” Affecting 300,000 newborns? In which time period?

 “This disease has 90% of mortality"; “important morbidity“; ”surgical correction is more difficult to resolve”; “The paraclinical diagnostic methods showed anomalous emergency of left main coronary artery from the pulmonary artery”; “physiopathology” - examples of incorrect use of the English language.

I have read this submission. I believe that I have an appropriate level of expertise to confirm that it is of an acceptable scientific standard, however I have significant reservations, as outlined above.

# Response

**Published:** 2016-07-26

**Authors:** Luis Gustavo Vilá Mollinedo

First of all, thank you for your time and recommendations.

 Addressing all this:

- We're presenting this approach as an alternative, also we think it's a bit superior having a coronary extension than a baffle procedure, due to the kinking or obstruction probability of these, but also just displaying what we did.
- I have changed the English use of all the terms and sentences you have proposed, although our English isn't great, we hope to have improved the readability.
- Finally it was our mistake, the sentence about the incidence should read "Affecting 1 of 300,000 newborns".

Thanks for your support, any other outlining would be appreciated.

# Peer review: Referee response for version 1

**DOI:** 10.5256/f1000research.9498.r15200

**Published:** 2016-07-25

**Authors:** Aiden Abidov

**Affiliations:** 1 Department of Medicine and Radiology, University of Arizona College of Medicine, Tucson, AZ, USA

A few minor things:

- Abstract: I recommend using a term "mild exertional dyspnea" instead of " little ...dyspnea".
- Figure 3: I would rather remove the Legend from the Figure (makes it less crowded).
- Figure 1 - would show a 3rd plate with a longitudinal view of the LM artery (if available) for a better understanding of the anatomy. Just an origin view is not that  impressive.

I have read this submission. I believe that I have an appropriate level of expertise to confirm that it is of an acceptable scientific standard.

# Response

**Published:** 2016-07-26

**Authors:** Luis Gustavo Vilá Mollinedo

First of all, thank you for your evaluation, really helpful, I changed the term of dyspnea for the one you suggested. As for the Figures, we have removed the legend, and as for the CT scan I do not have the one with the longitudinal view.

 Thanks for your help again.
